# Systemic delivery of TNF-armed myxoma virus plus immune checkpoint inhibitor eliminates lung metastatic mouse osteosarcoma

**DOI:** 10.1016/j.omto.2021.07.014

**Published:** 2021-08-06

**Authors:** John D. Christie, Nicole Appel, Hannah Canter, Jazmin Galvan Achi, Natalie M. Elliott, Ana Lemos de Matos, Lina Franco, Jacquelyn Kilbourne, Kenneth Lowe, Masmudur M. Rahman, Nancy Y. Villa, Joshua Carmen, Evelyn Luna, Joseph Blattman, Grant McFadden

**Affiliations:** 1 School of Life Sciences, Arizona State University, Tempe, AZ 85281, USA; 2 Biodesign Institute, Center for Immunotherapy, Vaccines and Virotherapy (CIVV), Arizona State University, Tempe, AZ 85281, USA; 3 Oncomyx Therapeutics, Phoenix, AZ 85004, USA

**Keywords:** myxoma virus, armed oncolytic virus, TNF-alpha, carrier cells, PBMCs, bone marrow, ICI, immune checkpoint inhibitors, K7M2, osteosarcoma

## Abstract

Solid cancers that metastasize to the lungs represent a major therapeutic challenge. Current treatment paradigms for lung metastases consist of radiation therapy, chemotherapies, and surgical resection, but there is no single treatment or combination that is effective for all tumor types. To address this, oncolytic myxoma virus (MYXV) engineered to express human tumor necrosis factor (vMyx-hTNF) was tested after systemic administration in an immunocompetent mouse K7M2-Luc lung metastatic osteosarcoma model. Virus therapy efficacy against pre-seeded lung metastases was assessed after systemic infusion of either naked virus or *ex vivo*-loaded autologous bone marrow leukocytes or peripheral blood mononuclear cells (PBMCs). Results of this study showed that the PBMC pre-loaded strategy was the most effective at reducing tumor burden and increasing median survival time, but sequential intravenous multi-dosing with naked virus was comparably effective to a single infusion of PBMC-loaded virus. PBMC-loaded vMyx-hTNF also potentially synergized very effectively with immune checkpoint inhibitors anti-PD-1, anti-PD-L1, and anti-cytotoxic T lymphocyte associated protein 4 (CTLA-4). Finally, in addition to the pro-immune stimulation caused by unarmed MYXV, the TNF transgene of vMyx-hTNF further induced the unique expression of numerous additional cytokines associated with the innate and adaptive immune responses in this model. We conclude that systemic *ex vivo* virotherapy with TNF-α-armed MYXV represents a new potential strategy against lung metastatic cancers like osteosarcoma and can potentially act synergistically with established checkpoint immunotherapies.

## Graphical abstract

**Figure:**
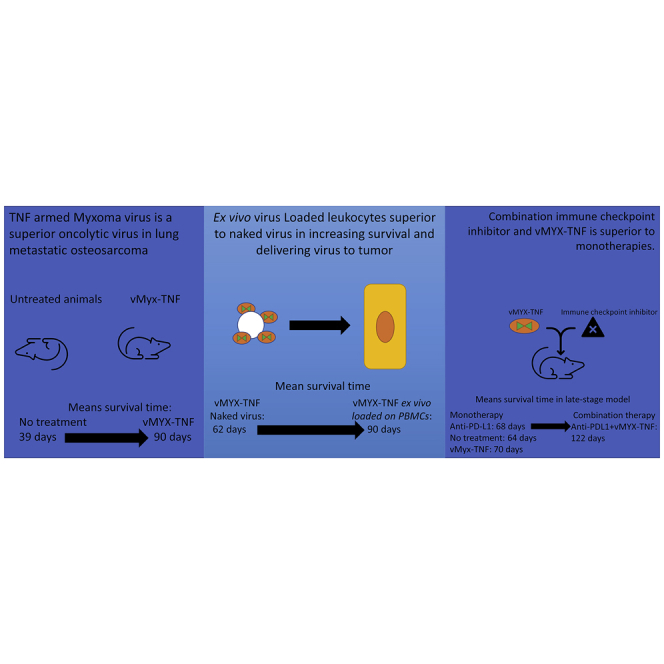

## Teaser

How to deliver and specific arming of oncolytic viruses are two of the biggest questions currently facing the field of oncolytic virotherapy. In this study, leukocytes are used to deliver TNF-α-armed myxoma virus to lung metastatic osteosarcoma tumors. The efficacy of these modalities is shown alone and in combination with immune checkpoint inhibitors.

## Introduction

Cancer is the current second leading cause of death in Americans today, killing almost 600,000 Americans in 2020.^1^^,^^2^ As of 2020, cancer was only second to heart disease as cause of death in the United States.^2^, ^3^, ^4^ While normally associated as a disease of advanced age, cancer is currently the fourth leading cause of death in adolescents and young adults.^5^^,^^6^ Although commonly grouped together, malignant neoplasms of younger populations and those of older populations differ extensively in their incidence rates, origin, development, mutation load, and their response to therapies.^7^^,^^8^ Osteosarcoma is one such cancer that is much more common in adolescents and young adults, but with a second peak in older populations.^9^^,^^10^ It is currently the eighth most common childhood malignancy and is poorly treated after it has metastasized out of the primary site in the bone. Osteosarcoma is the most common form of primary malignancy arising in the bones^11^^,^^12^ and develops from osteoblastic cells, especially during times of rapid bone growth, usually associated with the dysregulation of common tumor suppressors, including p53 and pRB.^9^^,^^13^^,^^14^ Although there is a total gross survival rate of 68% over 5 years, treatment, prognosis, and survival are still dependent on many factors.^13^ Current treatment strategies for osteosarcoma are highly dependent on the stage of the cancer at which the treatment is administered, with early intervention having positive outcomes; however, the prognosis for metastatic osteosarcoma is considerably worse, with the 5-year survival rate being 17% and the 10-year survival rate being 15%.^15^^,^^16^ Given the poor prognosis associated with later stage osteosarcoma, the development of new treatment strategies is necessary.

Recently, two new classes of cancer therapies have emerged as candidates to supplement current clinical strategies: immune checkpoint inhibitors (ICIs) and oncolytic virotherapy.^17^^,^^18^ Previous research has shown that therapeutic blockade of PD-1/PD-L1 signaling significantly increases survival time of immunocompetent mice inoculated systemically with syngeneic murine K7M2 osteosarcoma tumor cells, which mostly become localized within metastatic lung lesions after systemic intravenous (i.v.) infusion.^19^^,^^20^ Furthermore, the combination of anti-PD-L1 + anti- cytotoxic T lymphocyte associated protein 4 (CTLA-4) leads to both increased survival times and induces long-term tumor regression in 50% of the population, featuring acquired resistance to cancer re-challenge.^19^ However, this combinatorial ICI regimen is not without the potential for toxicity and has only shown 50% efficacy in the K7M2 model, and thus further advances are necessary to properly address metastatic disease in the lung.

With the movement toward designing more targeted therapies to treat cancer, and the growing realization that immunotherapy with ICIs alone will be insufficient to tackle many cancers adequately, oncolytic virotherapy has re-emerged as a co-treatment strategy that has the potential to convert as least some cancers from the ICI-resistant status to ICI-responsive.^19^^,^^21^^,^^22^ Although oncolytic virotherapy as an idea dates back over a century, it has only been in the past few decades that it has become a feasible modality for the treatment of cancers refractory to standard monotherapies, including ICIs.^22^^,^^23^ Oncolytic virotherapy exploits live viruses that have been either engineered or selected for their ability to selectively infect, replicate within, and kill cancer cells within tumor beds. Furthermore, these anti-cancer therapeutic capacities are augmented by the ability of oncolytic virus (OV) infection within the tumor bed to activate the host immune system against not only non-self antigens from the virus, but also self-derived tumor antigens or mutated neoantigens.^24^^,^^25^

Myxoma virus (MYXV) is well suited as a platform therapeutic virus for solid cancers such as osteosarcoma because of its unique biology. MYXV is a member of the family Poxviridae and the genus *Leporipoxvirus*.^24^^,^^25^ In nature, this virus is rabbit-specific and does not cause infection or disease in any non-lagomorph, including humans or mice. However, because of idiosyncrasies of carcinogenesis and the selection of progressively malignant phenotypes, most cancer cells invariably lose elements of their innate immune ability to resist infection by many viruses, including MYXV.^24^, ^25^, ^26^ Most human or mouse cancer cells behave phenotypically more like permissive rabbit cells, such that MYXV can selectively infect and kill them while leaving normal somatic cells and tissues unharmed. Another key feature of the biology in this virus system is the large and genetically stable double-stranded DNA (dsDNA) genome.^26^ Viral genes that regulate different forms of immune modulation and/or cell death can be easily knocked out and, in addition, therapeutic anti-cancer transgenes can be introduced as genetic knock-ins to either induce increased immune responses directed against tumor antigens and/or induce preferred pro-immunogenic forms of cancer cell death. To date, MYXV has been assayed *in vitro* in dozens of different human or murine tumor cell lines and been investigated *in vivo* in multiple murine models of solid or hematopoietic cancer.^26^, ^27^, ^28^ Finally, one unique advantage of MYXV is that this virus has evolved in nature to spread throughout the host via circulating infected leukocytes of many classes, and we have previously shown that *ex vivo* infection of either human or mouse primary leukocytes derived from the periphery or the bone marrow (BM) provides a convenient “carrier cell” strategy to deliver the virus to disseminated cancer sites available to leukocyte trafficking to sites, such as in the BM and spleen in the case of multiple myeloma.^29^, ^30^, ^31^ In this study, we explore the capacity of the systemic *ex vivo* delivery strategy of MYXV to treat metastatic osteosarcoma localized mostly in the lung.

One area of current interest is the addition of immune-enhancing transgenes into oncolytic viruses to further stimulate anti-tumor responses. In this regard, tumor necrosis factor alpha (TNF-α; hereafter referred to as TNF) is a cytokine that is an integral part of both the early inflammatory response and the acquired cellular immune response.^32^, ^33^, ^34^ Although preliminary experiments with systemic injections of soluble TNF ligand in mice initially seemed to back up this optimism, the transition from the lab to the clinic showed that TNF was not only a powerful activator of the immune system, but it also caused severe toxicities in patients treated systemically with the soluble ligand. Studies also showed that systemic TNF treatment did not induce the dramatic anti-tumor effects in patients that had been observed in murine models.^35^^,^^36^ Current research is ongoing to investigate how to target and/or express TNF more selectively within the tumor bed, thus hopefully ameliorating past observed severe systemic side effects.^37^ One way to achieve this is through the expression of TNF from an oncolytic virus that is restricted to tumor sites, such as MYXV.

In this study, we assessed MYXV armed with human TNF (vMyx-hTNF) in the K7M2-Luc (luciferase-expressing murine osteosarcoma K7M2 cells derived from the BALB/c lineage) syngeneic lung metastatic osteosarcoma model in BALB/c mice. Our preliminary studies indicated that unarmed MYXV was insufficiently therapeutic in this model, and hence we screened various MYXV constructs expressing immune-enhancing cytokines, which identified TNF-armed MYXV as a potential candidate for further testing. Human TNF is bioactive in the murine model and has the technical advantage that it can be distinguished from endogenous murine TNF that would likely be induced during therapy. We compared and contrasted three systemic delivery strategies involving i.v. infusion of either free (“naked”) virus, or else *ex vivo* loading of virus onto two different populations of carrier leukocytes, namely autologous BM leukocytes or autologous peripheral blood mononuclear cells (PBMCs), to administer TNF-armed MYXV after the osteosarcoma has been seeded into lungs. Finally, we combined vMyx-hTNF with three test ICIs in combination therapies to show that TNF-armed MYXV can potentially synergize with standard immunotherapy to treat and even eliminate lung metastatic osteosarcoma. The results of this study demonstrate the outstanding potential of TNF-armed MYXV as an oncolytic therapy for lung metastatic tumors, the use of mixed leukocyte carrier cells to improve the efficacy of treatment, and the ability of TNF-armed MYXV to synergize with approved ICIs.

## Results

### *Ex vivo*-loaded BM leukocytes armed with TNF-armed MYXV is therapeutic for lung metastatic syngeneic mouse osteosarcoma

**Figure 1:**
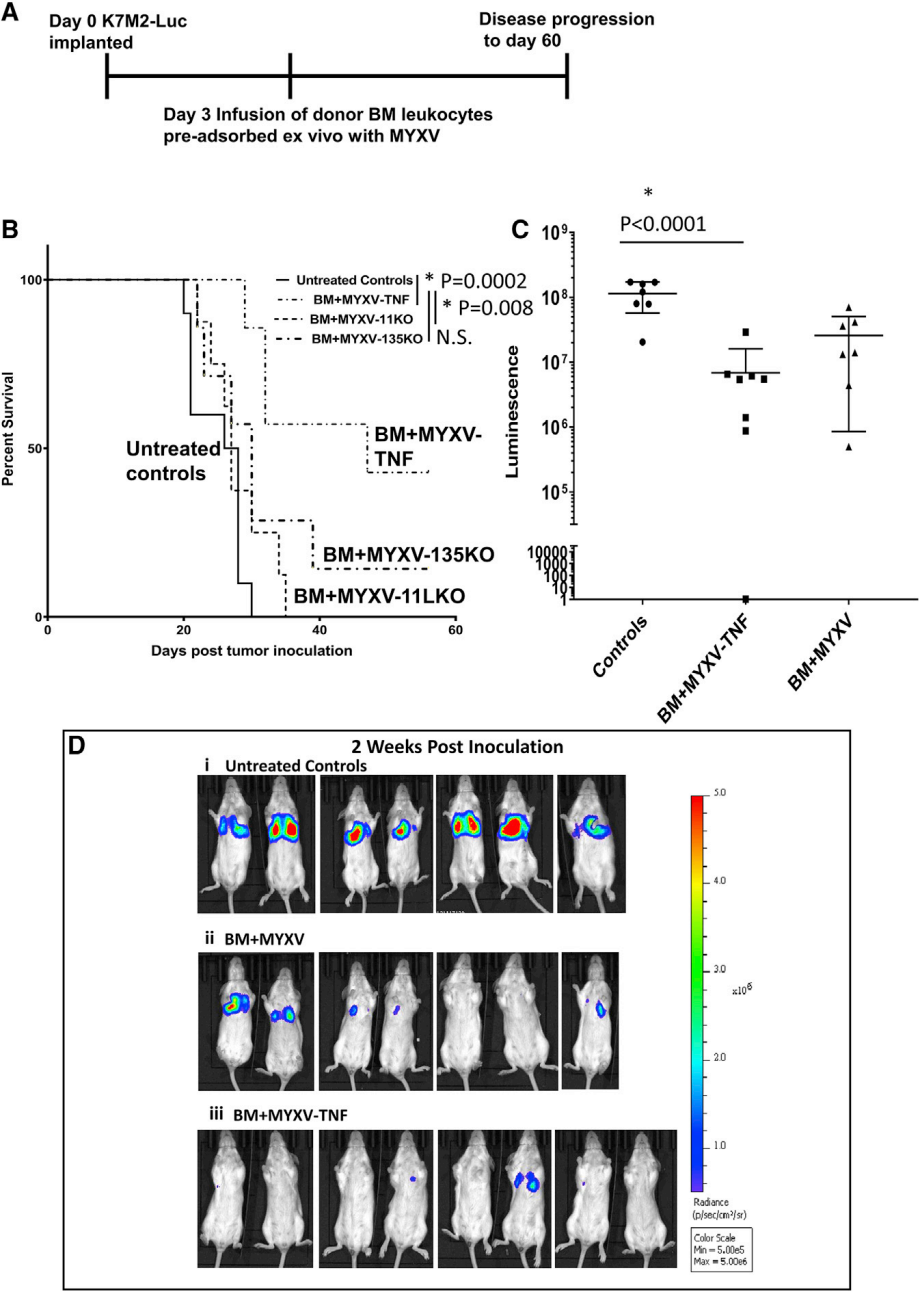
Systemic delivery of donor bone marrow (BM) leukocytes *ex vivo* loaded with TNF-armed MYXV increases mean survival and reduces tumor burden in mice with K7M2-Luc lung metastatic osteosarcoma (A) Diagram of experimental setup. K7M2-Luc cells implanted at day 0 via tail vein intravenous (i.v.) injection into BALB/c mice. Single dose treatment with test virus pre-loaded *ex vivo* on BALB/c BM leukocytes was at 3 days after tumor inoculation. Animals were then followed for 60 days. (B) Kaplan-Meier survival curves of animals treated with BM *ex vivo* loaded with either armed vMyx-hTNF (n = 8) (MYXV-TNF) or unarmed MYXV gene knockouts (vMyx-11KO [n = 8] or vMyx-135KO [n = 7]). Mice treated with vMyx-hTNF survived significantly longer than did animals treated with unarmed viruses or that were left untreated (n = 7). (C) Representative luminescence signals from K7M2-Luc-induced lung metastases at 2 weeks after tumor inoculation. Data shown (mean±SD) for unarmed BM+MYXV are for vMyx-135KO. (D) Representative luminescence images from K7M2-Luc-induced lung metastases at 2 weeks after tumor inoculation. (i) Control animals that received no treatment at 2 weeks after tumor inoculation. (ii) Animals that received BM *ex vivo* loaded with unarmed vMyx-135KO treatment (BM+MYXV) at 2 weeks after tumor inoculation. (iii) Animals that received BM *ex vivo* loaded with vMyx-hTNF (BM+MYXV-TNF) treatment at 2 weeks after tumor inoculation.

Previous studies performed in our lab demonstrated that the use of autologous BM leukocytes *ex vivo* pre-loaded with MYXV was the most effective delivery strategy for treating pre-seeded syngeneic multiple myeloma located in the BM and spleen.^31^ However, to date, no solid tumors have been tested as a target for MYXV virotherapy using this delivery method. To assess whether systemic delivery could be an effective strategy against lung metastatic tumors, the syngeneic luciferase tagged K7M2 murine lung metastatic osteosarcoma was chosen as a model. BALB/c mice were i.v. injected with 2 × 10^6^ K7M2-Luc osteosarcoma cells via the lateral tail vein. Essentially all subsequent tumors were detected in the lung, except for the occasional tail tumor vein tumors that tended to progress rapidly and had to be removed from the cohorts due to Institutional Animal Care and Use Committee (IACUC) regulations. Tumors were allowed to engraft in the recipient lungs for 3 days before animals were treated with 2 × 10^6^ BM cells *ex vivo* pre-loaded for 1 h with one of three test viruses: vMyx-M135 knockout (M135KO), vMyx-M11 knockout (M11KO), or vMyx-hTNF (MYXV expressing human TNF) (all at a multiplicity of infection [MOI] of 10) (Figure 1A). The unarmed knockout vMyx-M135KO and vMyx-M11LKO viruses were selected for screening based on previous oncolytic efficacy results in other tumor models.^31^^,^^38^^,^^39^ vMyx-hTNF was selected for screening because we hypothesized that a generalized immune-enhancing cytokine ligand such as TNF, when preferentially expressed in the context of a tumor bed, might allow increased anti-tumor efficacy compared to unarmed viruses, while avoiding the toxic effects associated with systemic delivery of the purified cytokine. Results from this screen revealed that only animals treated with the TNF-expressing MYXV that had been *ex vivo* loaded onto BM leukocytes had a significantly longer mean survival time (47 days) compared to animals comparably treated with either of the unarmed knockout viruses (vMyx-M135KO at 30 days and vMyx-M11LKO at 27 days) or compared to animals that were left untreated (27 days) (Figure 1B). Animals were imaged using *in vivo* imaging system (IVIS) imaging for luciferase expression measured by luminescence during the course of the 60-day experiment as an approximate real-time indication of tumor progression. At 2 weeks after tumor inoculation, animals that were left untreated had significantly larger tumors than did animals systemically treated with vMyx-hTNF/BM leukocytes (Figure 1C), although there was a trend to lower luminescence values in the unarmed knockout MYXV-treated animals compared to untreated controls (Figures 1C and 1D). Thus, unlike our previously published cancer models where unarmed MYXV was demonstrably efficacious as an anti-cancer therapeutic,^31^^,^^38^^,^^39^ in this stringent metastatic lung osteosarcoma model, arming of MYXV with an immunostimulatory transgene was essential in order to demonstrate therapeutic efficacy.

### *Ex vivo*-loaded PBMCs pre-loaded with TNF-armed MYXV are comparably effective to BM leukocytes as carrier cells

**Figure 2:**
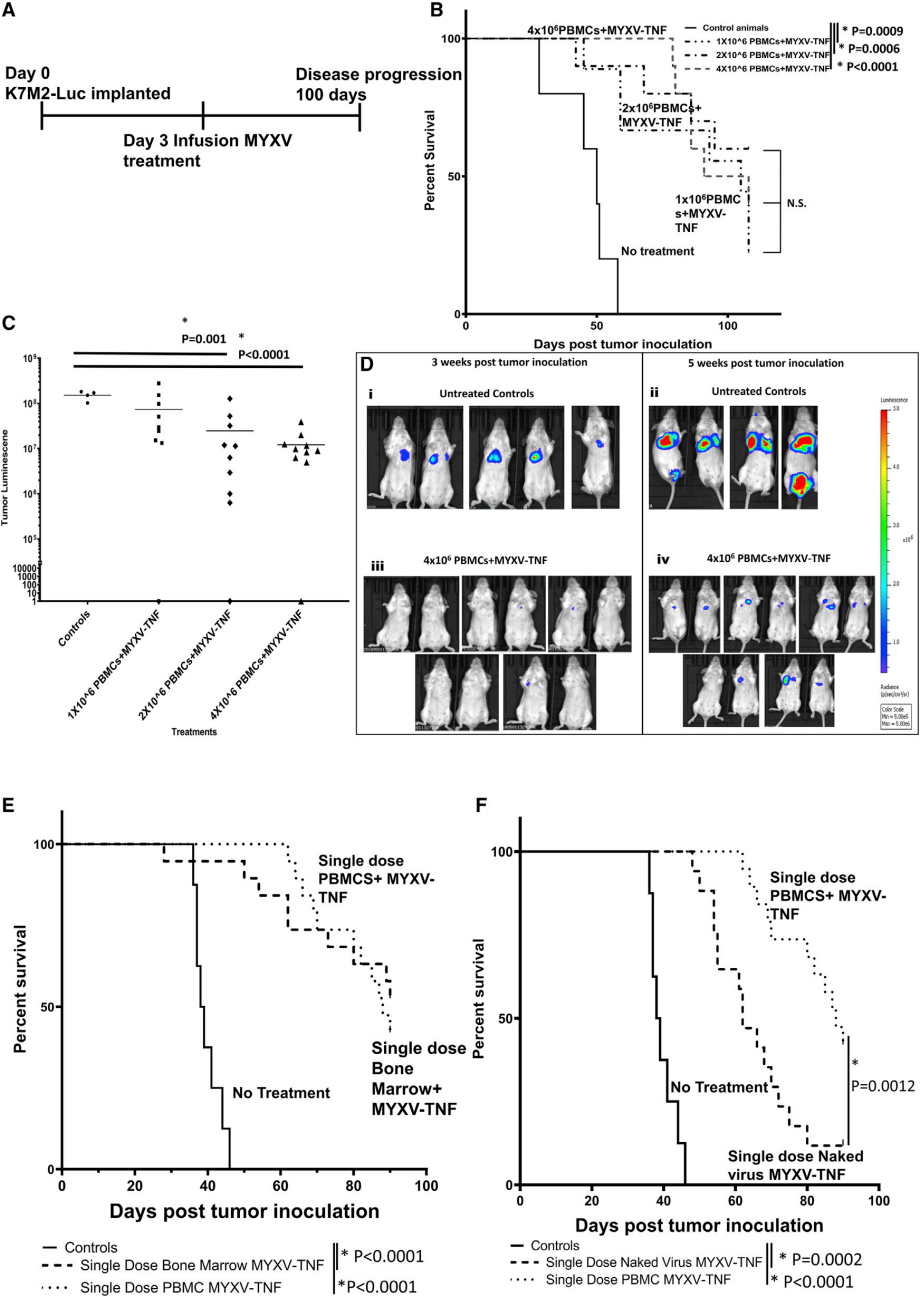
Systemic delivery of mixed leukocytes *ex vivo* pre-loaded with TNF-armed MYXV as compared to i.v. infusion of free virus (A) Diagram of experimental setup. BALB/c mice had K7M2-Luc cells implanted at day 0 via i.v. injection. Single dose systemic infusion with vMyx-hTNF (MYXV-TNF), either as free “naked” virus, after pre-loading *ex vivo* onto BALB/c PBMC carrier cells, or after pre-loading *ex vivo* onto BALB/c BM leukocytes was at 3 days after tumor inoculation. Animals were then followed for 100 days. (B) Kaplan-Meier survival curves of animals treated with three doses of PBMCs loaded *ex vivo* with vMyx-hTNF: 1 × 10^6^ (n = 10), 2 × 10^6^ (n = 10), and 4 × 10^6^ (n = 10) PBMCs, each loaded with an MOI of 10 ffu/cell each. Each cell/virus dose level treatment led to a similarly significant increase in mean survival time over untreated controls (n = 5). (C) Scatterplot showing individual luminescence signals at 5 weeks after tumor inoculation. Animals treated with 2 × 10^6^ and 4 × 10^6^ PBMCs loaded with vMyx-hTNF had significantly smaller tumor luminescence signals than did untreated animals. (D) Representative luminescence images from 2 and 5 weeks after tumor inoculation of animals left untreated (i and iii) or treated with 4 × 10^6^ PBMCs loaded with vMyx-hTNF. (E) Kaplan-Meier plot comparing a single dose of BM- versus PBMC-based *ex vivo* loading of vMyx-hTNF. Both treatments led to a significant increase in mean survival compared to untreated animals but were not significantly different from each other. (F) Kaplan-Meier plot comparing a single dose of PBMC-loaded vMyx-hTNF to a single systemic infusion of the identical dose (2 × 10^7^ infectious units) of free (naked) vMyx-hTNF. Both treatments led to a significant increase in mean survival time; however, the single dose PBMCs+vMyx-hTNF led to a significant further increase in mean survival time when compared to systemic i.v. infusion of a single dose of naked virus.

While the results with TNF-armed MYXV *ex vivo*-loaded BM leukocytes as carrier cells showed significant activity in reducing tumor burden and increasing overall survival time in mice compared to animals left untreated or treated with unarmed MYXV, autologous BM transplants are rarely used in the clinic for solid tumors. Because of this, autologous PBMCs were next tested as a potential carrier cell population against osteosarcoma tumors in the lung. Previously, in the case of pre-seeded multiple myeloma in the BM and spleen as the targets, we had observed that PBMCs were inferior as carrier cells of MYXV to mixed BM-derived leukocytes.^31^ As with the screening experiments using BM carrier cells described above, animals were inoculated at day 0 with K7M2-Luc cells, and 3 days after inoculation autologous donor PBMCs were harvested and *ex vivo* pre-loaded for 1 h with vMyx-hTNF, and then systemically infused into tumor-bearing animals at three different doses: 1 × 10^6^, 2 × 10^6^, and 4 × 10^6^ cells (all infected with an MOI of 10) (Figure 2A). Animals treated with vMyx-hTNF/PBMCs survived significantly longer than did control animals left untreated at all three doses of virus-loaded PBMCs (Figure 2B). Also, animals treated with 2 × 10^6^ and 4 × 10^6^ PBMCs loaded *ex vivo* with vMyx-hTNF had significantly lower tumor luminescence measurements compared to untreated animals at 5 weeks post-inoculation (Figures 2C and 2D). Once it was established that vMyx-hTNF-loaded PBMCs were effective at increasing mean survival of treated animals, they were compared to an identical dosing regimen with 2 × 10^6^ BM cells treatments. Animals systemically infused once with an identical dose of vMyx-hTNF (2 × 10^7^ infectious units of virus) delivered via either BM cells or PBMCs (2 × 10^6^ cells) both significantly increased mean survival time compared to untreated animals (mean survival 39 days) but were not significantly different from each other (mean survival of 88 days for vMyx-hTNF/PBMCs and 90 days for vMyx-hTNF/BM) (Figure 2E). However, while a single dose of systemically delivered free (naked) vMyx-hTNF did increase mean survival time compared to untreated controls, it was significantly less than for animals treated with vMyx-hTNF delivered by PBMCs as carriers (Figure 2F). Interestingly, four sequential systemic dosings with naked TNF-armed MYXV caused comparable anti-tumor efficacy to a single systemic dosing of vMyx-hTNF/PBMCs (Figure S2). Thus, *ex vivo*-loaded mixed leukocyte carrier cell delivery improves the anti-tumor efficacy of MYXV virotherapy compared to systemic infusion of naked virus in the metastatic lung osteosarcoma model, just as it does for multiple myeloma located in the BM.^31^

### Combination of systemic TNF-armed MYXV/PBMCs potentially synergizes with ICIs anti-PD-L1 or anti-PD-1 and induces long-term survivors

**Figure 3:**
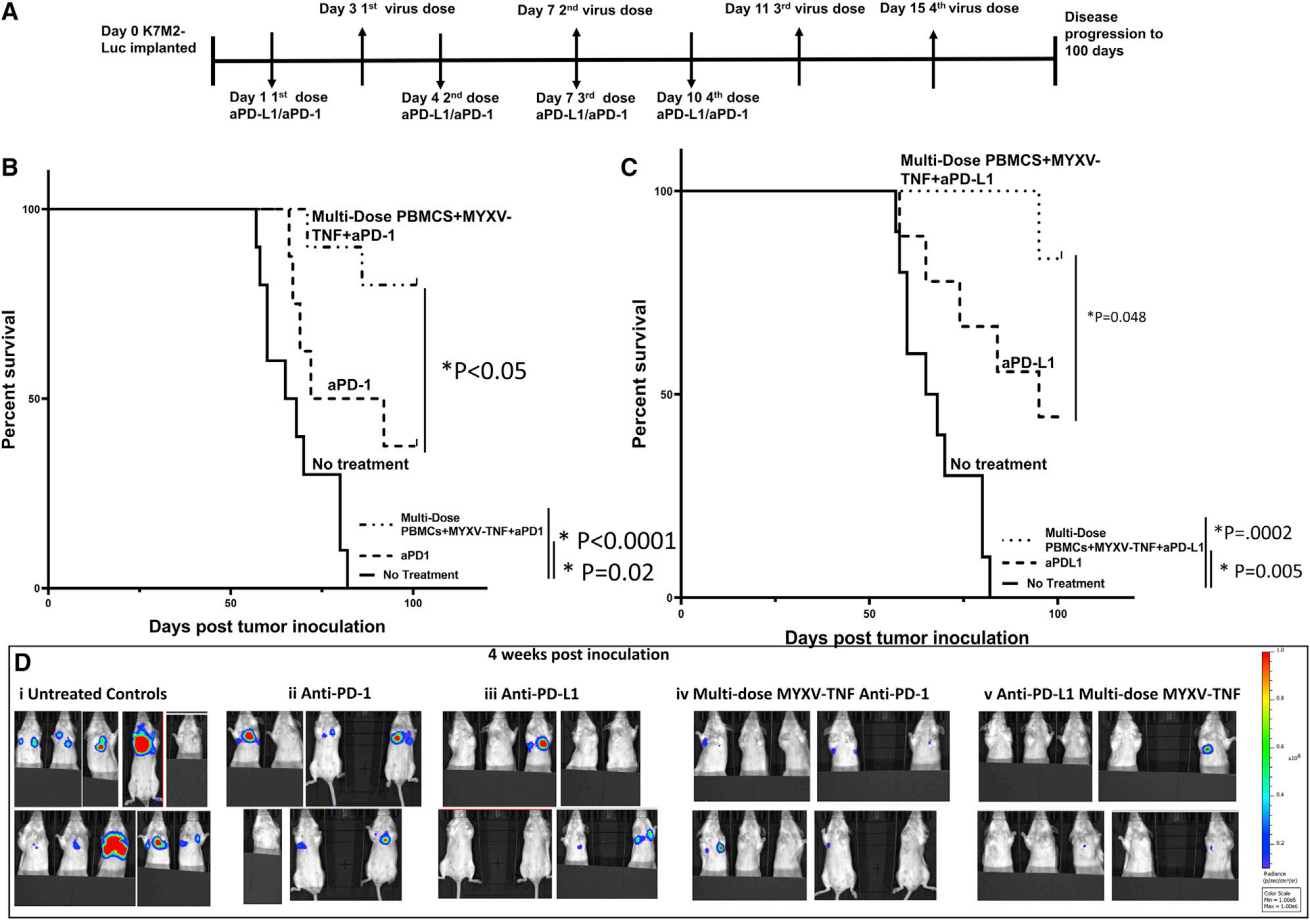
Combination therapy of systemic single dose TNF-armed MYXV/PBMCs plus ICI anti-PD-L1 or anti-PD-1 leads to increased survival and disease-free survivors compared to ICI monotherapy (A) Diagram of experimental setup. BALB/c mice were inoculated with K7M2-Luc cells at day 0 via i.v. injection. Treatment with ICIs started at day 1 after tumor inoculation and continued every third day for four total doses. Multi-dose treatments with vMyx-hTNF (MYXV-TNF) pre-loaded onto PBMC carrier cells started at 3 days after inoculation and continued every fourth day for a total of four doses. Animals were then followed for 100 days. (B) Kaplan-Meier survival curves comparing anti-PD-1 alone (n = 7) to a combination of vMyx-hTNF/PBMCs plus anti-PD-1 (n = 10). ICI monotherapy increased mean survival compared to untreated controls, and the combination therapy increased mean survival compared to ICI monotherapy. (C) Kaplan-Meier survival curves comparing anti-PD-L1 alone (n = 9) to a combination of vMyx-hTNF/PBMCs plus anti-PD-L1 (n = 10). ICI monotherapy increased mean survival compared to untreated controls, and the combination therapy further increased mean survival compared to ICI monotherapy. (D) Representative tumor luminescence images of animals at 4 weeks after tumor inoculation: (i) untreated control animals, (ii) anti-PD-1 alone, (iii) anti-PD-L1 alone (iv) combination therapy of vMyx-TNF/PBMCs plus anti-PD-1, (v) combination therapy of vMyx-TNF/PBMCs plus anti-PD-L1.

ICIs are becoming the standard of care for many cancers and are being tested in combination with multiple oncolytic viruses.^17^ Previous studies have shown that K7M2 tumors in the lung partially respond to the ICI anti-PD-L1, increasing mean survival time of animals receiving monotherapy.^20^ Because of this, we tested whether combining the established anti-PD-L1 therapy regimen in combination with vMyx-hTNF (MOI of 10) *ex vivo* loaded onto PBMCs would further increase mean survival time of animals compared to either therapy alone. As with other experiments, animals were pre-seeded in the lung with K7M2-Luc cells, defined as day 0. Animals were multi-dosed with vMyx-hTNF *ex vivo*-loaded PBMCs four times starting 3 days after tumor inoculation, then every 4 subsequent days, for a total of four sequential virus/PBMC doses. The animals were also treated with either anti-PD-1 or anti-PD-L1 per the dosing scheduled described in Lussier et al.,^20^ starting 24 h after tumor inoculations and continued every third day for a total of four doses (Figure 3A). As predicted, the ICI monotherapies both significantly increased mean survival compared to untreated animals (Figures 3B and 3C). Animals treated with the combination of ICI plus vMyx-hTNF/PBMCs significantly increased mean survival times compared to animals treated with ICI monotherapy (day 82 for anti PD-1 and day 92 for anti-PD-L1), such that animals treated with either of the combination therapies all exhibited greater than 50% long-term survival (Figures 3B and 3C). Imaging animals for luminescence as an approximate surrogate of tumor burden showed that all animals with combination therapy had little to no tumor luminescence at 4 weeks after tumor inoculation, compared to monotherapies where a subset of animals showed advanced tumors in each group (Figure 3D).

### Multi-dosed TNF-armed MYXV/PBMCs plus anti-PD-L1 combination therapy is therapeutic even in mice bearing established late-stage K7M2-Luc lung tumors

**Figure 4:**
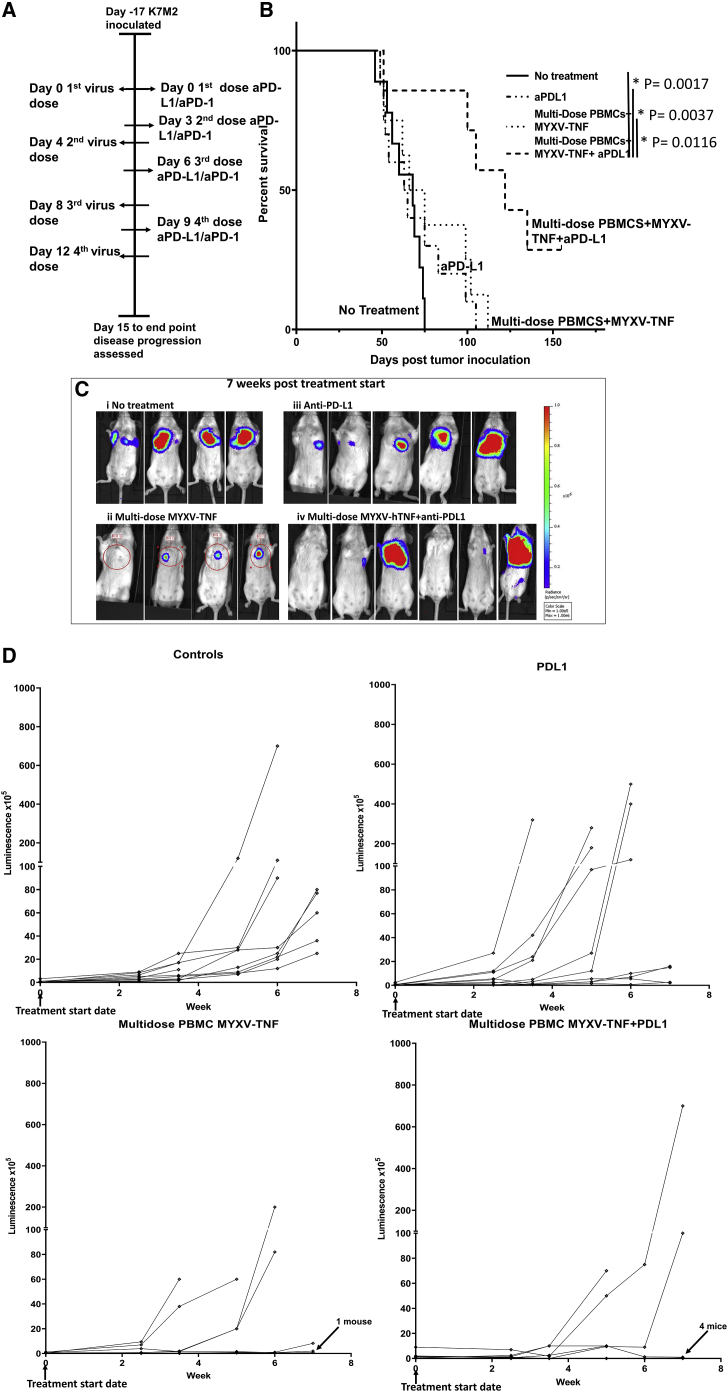
Combination therapy of animals bearing late-stage K7M2-Luc tumors with anti-PD-L1 plus multiple doses of TNF-armed MYXV/PBMCs leads to increased mean survival compared to monotherapies (A) Diagram of experimental setup. BALB/c mice were inoculated with K7M2-Luc cells at day −17 via i.v. injection of K7M2-Luc cells. Treatment with ICI started at treatment day 0 and continued every third day for four total doses. Multi-dose treatment with vMyx-hTNF (MYXV-TNF) using *ex vivo*-loaded PBMC carrier cells started at treatment day 0 and continued every fourth day for four total doses. Disease progression was then followed for 155 days. (B) Kaplan-Meier survival curves comparing untreated controls (n = 9), vMyx-hTNF/PBMC monotherapy (n = 8), anti-PD-L1 plus vMyx-hTNF/PBMC combination (n = 7), and anti-PD-L1 monotherapy (n = 10). Animals treated with combination therapy survived significantly longer than did animals left untreated, or with either of the monotherapies. (C) Representative luminescence images are shown at 7 weeks after tumor inoculation: (i) untreated controls, (ii) vMyx-hTNF/PBMCs (MYXV-TNF), (iii) anti-PD-L1 therapy, and (iv) vMyx-hTNF/PBMCs (MYXV-TNF) plus anti-PD-L1 combination therapy. (D) Line graphs showing individual tumor luminescence progression during the first 7 weeks after tumor inoculation.

The results showing that the combination of anti-PD-L1 plus vMyx-hTNF/PBMCs increased mean survival compared to monotherapy led us to ask whether this was an additive effect or whether this represented potential synergy between the two modalities. In order to test treatment of fully established late-stage lung tumors, implanted K7M2-Luc cells were allowed to develop into broadly luminescence-positive lung metastases for 17 days, as defined by allowing a threshold of average tumor luminescence to reach 5 × 10^5^ radiance units across all cohorts. At this point, control animals exhibit extensive lung disease and are uniformly within several weeks of the endpoint; furthermore, none of the monotherapies tested is efficacious at prolonging survival at this later stage of the model. At day 20 after tumor inoculation, which for this cohort is defined as experimental day 0, treatment with *ex vivo* virotherapy plus immune checkpoint therapy was initiated. Animals were treated with four sequential doses of anti-PD-L1 therapy four times every third day and with vMyx-hTNF (2 × 10^7^ infectious units) loaded *ex vivo* onto 2 × 10^6^ PBMCs every fourth day for four doses total (Figure 4A). Animals treated with either virus/PBMCs or ICI monotherapy alone showed no increase in mean survival compared to untreated animals (mean survival time: 68 days for untreated, 64 days for anti-PD-L1 alone, and 70 days for vMyx-hTNF/PBMCs). However, animals treated with combination anti-PD-L1 plus vMyx-hTNF/PBMCs showed a significant increase in mean survival time (to day 122) (Figure 4B). Tumor luminescence progression was followed during the course of the first 7 weeks of this study using IVIS imaging for luciferase expression from the tumor. Representative images are shown in Figure 4C, and progression in individual animals is shown in Figure 4D. Tracking of individual tumors showed that animals that were left untreated all had tumor signals that increased over time at different rates and all reached the endpoint by day 70. Animals treated with anti-PD-L1 or vMyx-hTNF/PBMCs alone had some animals with tumor luminescence readings that remained detectable but static during the course of the 7 weeks, and some that increased in luminescence during that period. The group treated with the combination of systemic vMyx-hTNF/PBMCs plus anti-PD-L1 manifested tumors that either reduced in luminescence or remained static in all but three of the animals. We conclude that once metastatic lung disease has progressed to a late stage with tumor signal detectable throughout the tissue, monotherapy with either ICI or TNF-armed MYXV alone is insufficient to halt the disease, but combinatorial use of multi-dose virus/PBMCs plus ICI is significantly therapeutic and can result in significant disease regression and long-term survival.

### Combination with the ICI anti-CTLA-4 also shows potential synergy with multi-dosed TNF-armed MYXV/PBMCs

**Figure 5:**
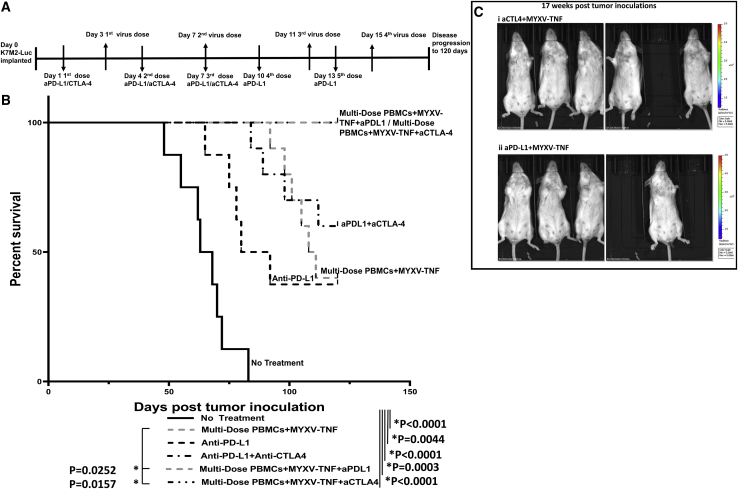
Combination anti-CTLA-4 or anti-PD-L1 plus multi-dose TNF-armed MYXV/PBMC virotherapy leads to long-term survival compared to monotherapies (A) Diagram of experimental setup. BALB/c mice were inoculated with K7M2-Luc cells at day 0 via i.v. injection. Treatment with ICIs started at day 1 after tumor inoculation and continued every third day for four total doses. Treatment with vMyx-hTNF (MYXV-TNF) using PBMC carrier cells started at 3 days post-inoculation and continued every fourth day for four total doses. Animals were then followed for 120 days. (B) Kaplan-Meier survival curves comparing monotherapy with multi-dose vMyx-hTNF/PBMCs (n = 10), anti-PD-L1 (n = 8), and combinations of anti-PD-L1/anti-CTLA-4 (n = 10), vMyx-hTNF/anti-PD-L1 (n = 6), and vMyx-hTNF/anti-CTLA-4 (n = 7). All therapies led to a significant survival increase compared to untreated controls (n = 8). Combination of vMyx-hTNF/PBMCs plus either anti-PD-L1 or anti-CTLA-4 increased mean survival and generated long-term survivors, as compared to vMyx-hTNF/PBMC or ICI monotherapies. (C) Representative tumor luminescence images at 17 weeks (119 days) after tumor inoculation showing animals treated with (i) vMyx-hTNF/PBMCs plus anti-PD-L1 and (ii) vMyx-hTNF/PBMCs plus anti-CTLA-4. These animals were tumor-free at the study end date of 120 days.

In previous experiments published in Lussier et al.,^19^^,^^20^ K7M2-induced lung metastases are partially responsive to the ICI anti-PD-L1, but not to anti-CTLA-4 as a monotherapy. However, when combined with anti-PD-L1, anti-CTLA-4 co-treatment generated long-term durable anti-tumor immunity in about 50% of mice. Based on these published results and our results above showing the potential for anti-PD-L1 to synergize with vMyx-hTNF/PBMC virotherapy, we next assessed whether anti-CTLA-4 would also work in combination with the virotherapy. To test this, animals were inoculated with K7M2-Luc at day 0, and then ICI (either anti-PD-L1 or anti-CTLA-4) was administered every 3 days subsequent to the first dose: anti-PD-L1 was administered for five total doses, starting at day 1 post-implantation, whereas anti-CTLA-4 was administered only for three total doses (due to the toxicity of this ICI). vMyx-hTNF (2 × 10^7^ infectious units) *ex vivo* loaded on 2 × 10^6^ PBMCs was systemically administered every 4 days subsequent to the first dose for four total doses (Figure 5A). Results from this study showed that animals treated with anti-CTLA plus vMyx-hTNF/PBMCs were essentially identical to animals treated with anti-PD-L1 plus vMyx-hTNF/PBMC combination therapy, with 100% of animals in both cohorts surviving out to at least 100 days after tumor inoculation (Figure 5B). Animals in both ICI plus MYXV combination therapy groups remained healthy and free of detectable tumor signal past 100 days after tumor inoculation as assessed by tumor luminescence (representative image shown in Figure 5C) and were considered long-term survivors.

### Systemic treatment with vMyx-hTNF/PBMCs induces unique signature cytokine upregulation mediated by the virus platform and by the TNF ligand

TNF is a powerful activator of the innate and adaptive immune system, through direct activation and also through downstream stimulation of many diverse subsets of immune cells. In part, this secondary activation is mediated by the induced expression of secondary cytokines upregulated in response to primary stimulation by TNF. We hypothesized that K7M2-bearing animals treated with vMyx-hTNF/PBMCs would induce a unique cytokine expression profile compared to tumor-bearing recipients either left untreated or treated with unarmed wild-type MYXV. In order to test this, animals were inoculated with K7M2-Luc cells on day 0. At 3 days after tumor inoculation, animals were left untreated or were treated with either vMyx-hTNF or vMyx-GFP (a wild-type reporter MYXV expressing EGFP) each loaded *ex vivo* onto 2 × 10^6^ PBMCs at an identical MOI of 10. At 1, 3, and 24 h after systemic virus/PBMC infusion, blood was collected and serum was separated and then assessed for altered circulating cytokine levels by an ELISA-based multiplex cytokine analysis. Results were then segregated into changes in cytokine expression based on the treatment group; that is, changes associated with unarmed MYXV, changes in TNF-armed MYXV, and finally induced changes that were comparable for both viruses. To do this, cytokine modulations by each of the virus treatments were compared to untreated animals for significant change, and then to each other.

**Figure 6:**
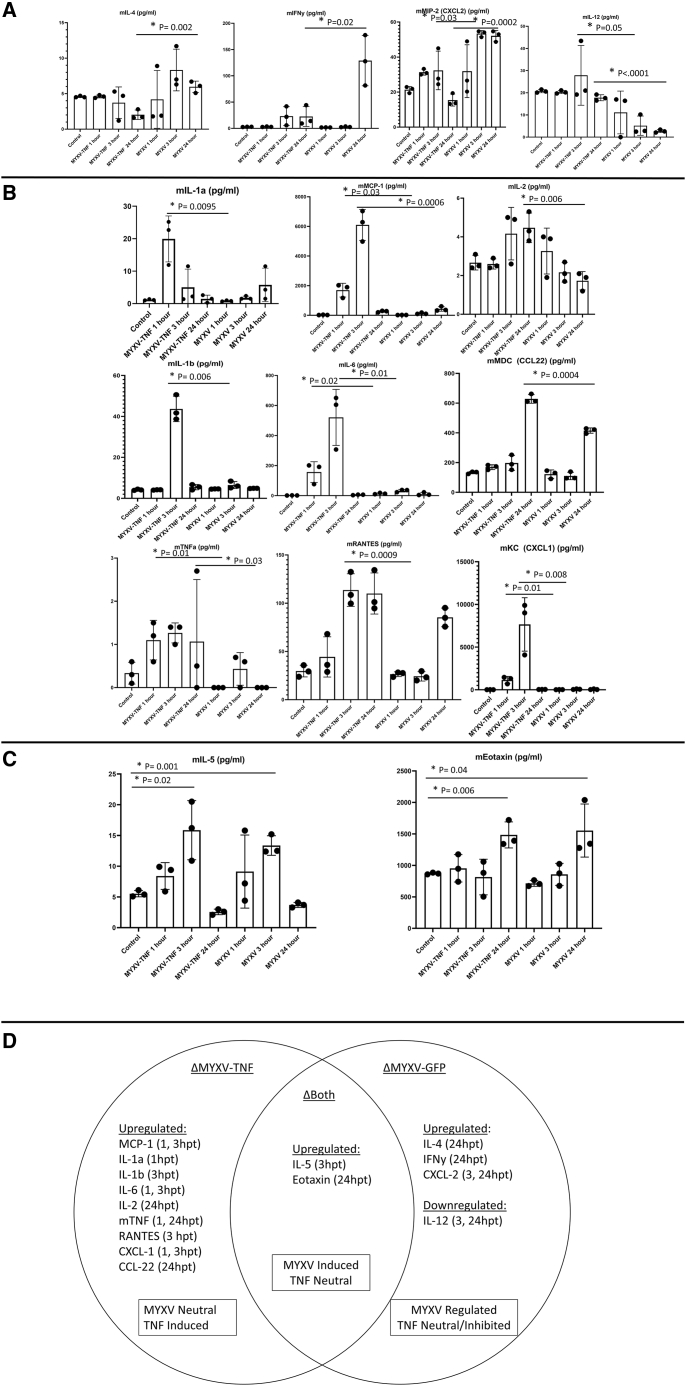
Circulating cytokine analysis of tumor-bearing animals early after treatment with TNF-armed MYXV/PBMCs show unique signatures induced by the unarmed MYXV platform and by the TNF transgene BALB/c mice were inoculated with K7M2-Luc cells at day 0. At day 3, animals were either left untreated or treated with unarmed vMyx-GFP/PBMCs (MYXV) or armed vMyx-hTNF/PBMCs (MYXV-TNF). Serum was collected at 1, 3, or 24 h after virus treatment. (A) Circulating cytokines in the MYXV-treated groups that were altered when compared to both untreated controls and vMyx-hTNF/PBMCs. Note that three cytokines were upregulated by the MYXV platform alone (mIL-4, mIFNγ, and mMIP-2/mCXCL2) and one was downregulated (mIL-12) (mean±SD). (B) Cytokines in the vMyx-hTNF/PBMC cohort that were upregulated when compared to both untreated and unarmed MYXV groups. (C) Cytokines that were similarly upregulated by both TNF-armed and unarmed MYXV groups compared to the untreated controls. (D) A Venn diagram showing cytokines that were modulated in TNF-armed MYXV, unarmed MYXV, or both. Cytokines were assigned as MYXV-neutral/TNF-induced, MYXV-induced/TNF-neutral, and MYXV-regulated/TNF-regulated.

In the first group were cytokines that were upregulated or downregulated in the unarmed control MYXV cohort compared to untreated controls or TNF-armed MYXV-treated animals (Figure 6A). Cytokines murine interleukin-4 (mIL-4) and murine interferon γ (mIFNγ) expression levels were both uniquely induced at 24 h with unarmed MYXV treatment compared to either control animals or those treated with TNF-armed MYXV. At both 3 and 24 h, the unarmed MYXV-treated animals also exhibited increased expression of murine (m)CXCL2 (mMIP-2) and decreased expression of mIL-12 compared to TNF-armed MYXV. We conclude that these four cytokines were each modulated by both the TNF transgene and by the MYXV platform.

The second set included nine cytokines that were upregulated uniquely in the TNF-armed cohort, as compared to untreated controls but were not upregulated in the unarmed MYXV cohort (Figure 6B). In this “TNF-specific” group, all nine cytokines were significantly upregulated compared to the unarmed MYXV or the untreated controls. At the earliest (1 h) time point, mIL-1α was the first TNF-upregulated cytokine to be selectively detected, and this upregulation ceased by 3 h, whereas at both the 1- and 3-h time points, mMCP-1 (mCCL-2), mIL-6, and mKC (mCXCL-1) were all increased. Beginning at 3 h post-treatment, mIL-1β and mRANTES (mCCL5) were both upregulated in the TNF-only cohort. By 24 h, mIL-2 and mMDC (CCL22) were selectively increased in the TNF-armed MYXV group. Finally, since any murine TNF that is produced endogenously and human TNF expressed by the viral transgene can be distinguished by ELISA, the host-derived mTNF was induced early at the 1- and 24-h time points only in the vMyx-hTNF set.

In the final group (Figure 6C) were cytokines that significantly increased in both virus treatment groups compared to the untreated controls but were not affected by the TNF transgene expression. In this group, two cytokines were upregulated, at 3 h mIL-5 was increased and at 24 h mExotaxin was increased after either virus treatment compared to untreated controls. We conclude that both the unarmed MYXV platform and the TNF-expressing MYXV each contributed to rapid innate cytokine responses soon after systemic virus dosing with *ex vivo*-loaded PBMCs in tumor-bearing mice, but that the TNF transgene uniquely induced both upregulation and downregulation of key host cytokines that can be correlated with improved anti-tumor efficacy (Figure 6D).

### Expression of transgene and trafficking of myxoma is dependent on PBMC loading and tumor status

**Figure 7:**
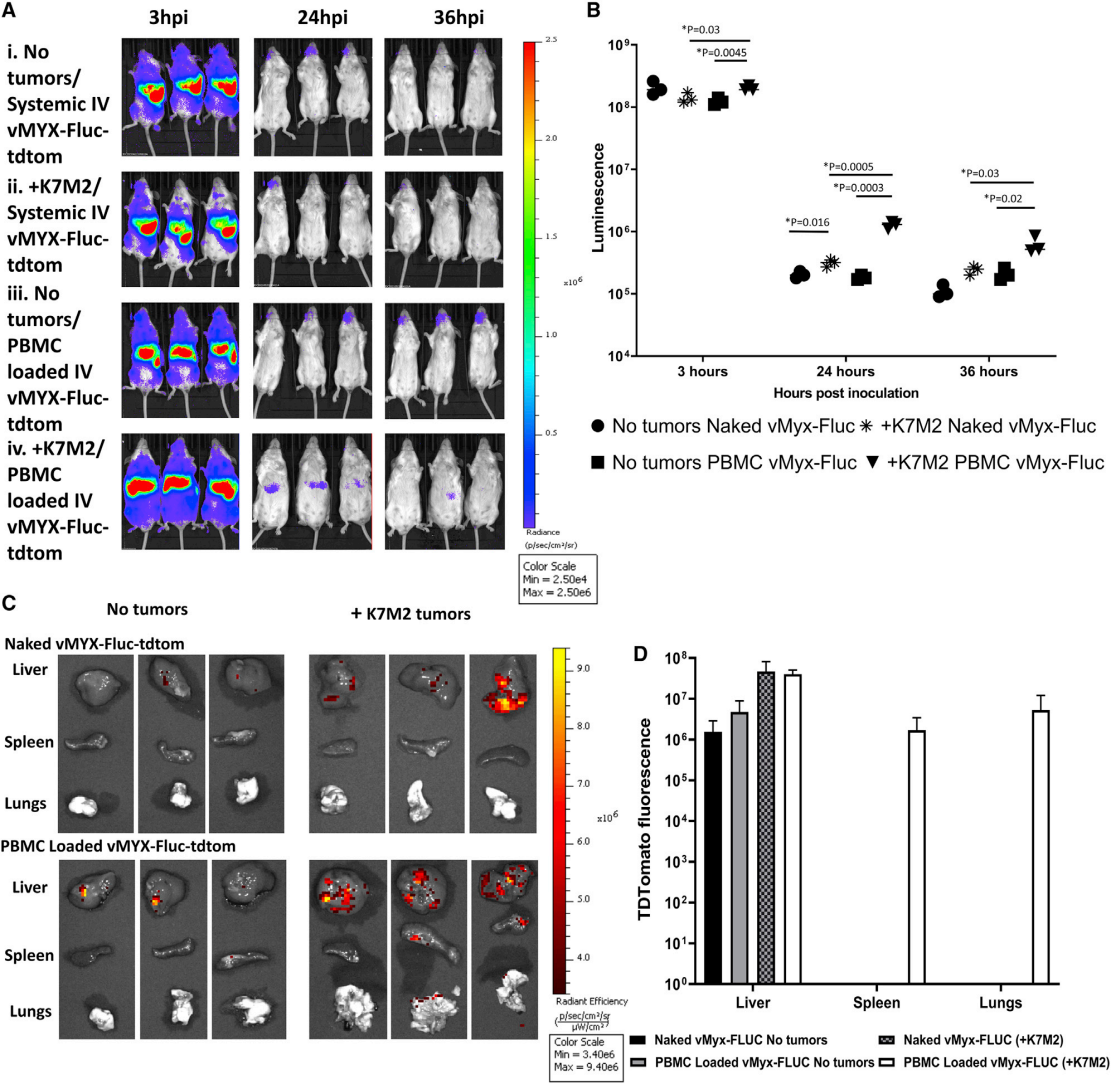
PBMCs loaded *ex vivo* with myxoma virus express increased levels of transgene in the periphery and more efficiently traffic to K7M2-bearing lungs than does systemically infused naked virus BALB/c mice bearing untagged K7M2 tumors seeded 25 days earlier, or control mice with no tumors, were treated with vMyx-Fluc-tdTomato (vMyx-Fluc-tdTom) and imaged after viral inoculation (hpi) for virally expressed luciferase at 3, 24, and 36 h. The Fluc transgene was expressed under a viral early/late promoter (i.e., expressed in peripheral leukocytes), whereas the tdTomato transgene was expressed under a late promoter and thus restricted to K7M2 tumor cells. At 36 hpi animals were euthanized and organs (liver, spleen, and lungs) were imaged for tdTomato fluorescence. (A) Images showing 3, 24, and 36 hpi luciferase luminescence from vMyx-Fluc-tdTom. (B) Scatterplot showing discrete luminescence data from each animal at 3, 12, and 24 hpi. (C) Images of excised organs from animals shown in (A) at 36 hpi. Tumors were imaged using IVIS imaging for tdTomato expressed (in K7M2 tumor cells) by vMyx-Fluc-tdTom in liver, spleen, and lungs. (D) Bar graph showing average fluorescence in liver, spleen, and lungs from animals with and without tumors and treated systemically with naked virus or PBMCs loaded *ex vivo* with vMyx-Fluc-tdTom (mean±SD).

The question of how do PBMCs mediate increased efficacy compared to systemically infused naked virus is an important one. Two major potential mechanisms for this include the ability to produce more of the efficacious transgenes, and the ability to deliver more virus to the tumor bed. To test this, animals were either inoculated with untagged K7M2 cells at day 0 or left tumor free. At 25 days after tumor inoculation, animals were treated either with systemic naked virus infusion or with vMyx-Fluc-tdTomato (vMyx-Fluc-tdTom) loaded on PBMCs. Starting at 3 h post-viral infusion (hpi) animals were imaged using IVIS imaging for luciferase luminescence until 36 hpi. At 36 hpi animals were euthanized and livers, spleens, and lungs were excised and imaged for tdTomato fluorescence. Results from this experiment showed that the peak luminescence was at 3 hpi and reduced over time until 36 h. Animals with K7M2 tumors and that were treated with PBMCs loaded with vMyx-Fluc-tdTom showed the highest average peaks at 24 and 36 hpi, and they had a significantly higher average luminescence compared to animals with K7M2 treated with naked virus vMyx-Fluc-tdTom at all three time points (Figures 7A and 7B). Furthermore, when organs were excised and imaged for tdTomato expression, tdTomato was only found in the lungs of K7M2 tumor-bearing animals, which were treated with PBMCs loaded with vMyx-Fluc-tdTom (Figures 7C and 7D). This is evidence that loaded PBMCs not only are able to better allow for sustained transgene expression in tumor bearing animals, but also that PBMCs are better able to deliver virus to the tumor bed.

## Discussion

Lung metastatic osteosarcoma, similar to many other types of lung metastatic cancer, represents a major clinical challenge. When diagnosed early, treatment of primary osteosarcoma tumor bone lesions leads to a 5-year survival rate >70%, but once the tumor has metastasized to the lung, 5-year survival rates drop to 20%. Previous research has shown that deactivation of the suppressive features of the acquired immune system via immune checkpoint inhibition is a potential new therapy.^19^^,^^20^ Oncolytic viruses have been shown in a wide variety of tumor types to both activate the immune response on their own and can function synergistically with therapies such as ICIs.^23^ For oncolytic viruses capable of genetic engineering to allow insertion of ectopic transgenes, the capacity to arm the virus with additional immune-enhancing regulators is a genuine therapeutic advantage. The issue of which transgenes to choose that can upregulate the innate and acquired immune system in order to bolster anti-tumor immune responses from an infected tumor bed is a topic of widespread interest. In this regard, TNF is a powerful activator of both the early innate and later adaptive immune responses, but therapeutic use of this cytokine has been hampered by the clinical experience that systemic treatment with TNF has been linked to many undesirable side effects.^40^ It is predicted that expressing TNF locally from an oncotropic virus such as MYXV, which can only productively propagate in tumor cells, and thus limit the induced level of circulating TNF, will lower the risk associated with systemic TNF treatment while hopefully preserving its anti-cancer properties.

The ability to combine oncolytic viruses with ICIs is a major area of study at present. ICI monotherapy generally requires a previously stimulated acquired immune response to tumor antigens that has become suppressed within the tumor bed, and ICIs are not by themselves thought to induce novel anti-tumor immune responses *de novo*. Thus, co-therapies capable of generating new anti-tumor immune responses in ICI-nonresponsive cancers are needed. Oncolytic viruses, when they are appropriately delivered to the target tumors, are thought to be capable of turning immune cold tumors hot by generating novel immune responses against both the virus and tumor antigens that become exposed within the virus-infected tumor bed. This property can be coupled with the documented ability of ICIs to circumvent the tumor-driven suppression of the immune response and offers a unique way to synergize these two therapies in the treatment of ICI-resistant cancers.^41^ The data presented herein show that TNF-armed MYXV, as systemically delivered via mixed carrier cells, such as PBMCs, in combination with ICIs such as anti-CTLA-4, anti-PD-1, or anti-PD-L1 can lead to long-term disease-free survival in animals when treatment is initiated early after tumor implantation in the lung. Importantly, the combination of anti-PD-L1 plus multi-dosed systemic TNF-armed MYXV/PBMCs can significantly increase mean survival even when lung tumors have progressed to the late stage where monotherapies all fail. The ability to use virus plus ICI combination treatments at late-stage metastatic lung disease progression when monotherapies fail represents evidence of potential synergy between ICIs such as anti-PD-L1 and TNF-armed MYXV. Previous experiments using this K7M2 metastatic osteosarcoma model have shown that low-dose anti-CTLA-4 can synergize in combination with anti-PD-L1 therapy to confer some level of durable tumor-free survival, but it is completely ineffective as a monotherapy in this model.^19^ Our data presented herein show that systemic administration of vMyx-hTNF/PBMCs, in combination with either low dose anti-CTLA-4 or standard dose anti-PD-L1, significantly increases the mean survival compared to the virus monotherapy and can generate 100% disease-free survivors. Given the broad range of side effects associated with combinatorial ICIs, ranging from mild to life threatening, the ability to replace one of the ICIs with an oncolytic virus could serve to limit side effects by reducing the ICI dosages needed for therapeutic effect.^42^^,^^43^

Delivery of oncolytic viruses to sites of metastatic cancer, such as in the lung, is a major challenge. To our knowledge, this is the first study to combine the use of autologous PBMCs or BM leukocytes as carrier cells to facilitate armed oncolytic virus therapy of lung metastases. Previous work in our lab has shown that mixed leukocytes, when pre-loaded *ex vivo* with MYXV, can act as effective carriers for treatment of murine multiple myeloma located in the BM and spleen; however, no study to date has tested this systemic carrier cell delivery modality for MYXV to target metastatic solid tumors.^31^ Carrier cell delivery is a major area of study in the oncolytic virus field because it can increase the effective dosing of virus in hard-to-reach tumor sites and offers the potential to bypass circulating immune barriers. This dual ability to potentially deliver more virus to the tumor bed and at the same time circumvent viral clearance elements of the immune system that can compromise delivery, such as antibodies and complement, is very attractive.^44^ The natural biology of MYXV makes it well suited for carrier cell delivery, naturally disseminating in rabbit hosts via circulating leukocytes. The data in this study show that MYXV can be loaded *ex vivo* onto mixed leukocytes derived from either BM or PBMCs and can achieve improved survival results compared to the same dose of free naked virus delivered systemically through i.v. administration. Importantly, four sequential i.v. doses of naked virus can generate comparable efficacy to that of a single dose of TNF-armed MYXV loaded on PBMCs, suggesting that systemic administration with either multi-dosed naked virus or single-dosed *ex vivo* leukocyte-bound virus can each have clinical applications. When looking specifically at trafficking and viral transgene expression, the difference between modalities becomes even clearer. Early transgene expression in tumor-bearing animals treated with MYXV/PBMCs was significantly higher than those treated with systemic naked virus infusion. Furthermore, at the termination of the experiment, only in tumor-bearing animals were excised lungs found to be expressed tdTomato, which is driven by the poxvirus late promoter P11. This is direct evidence of tumor-specific delivery of MYXV by PBMCs, which was not found in systemic naked virus delivery, or in the absence of tumors. Indeed, it is likely that infusion of free virus simply results in less efficient binding to circulating PBMCs than in the *ex vivo* preloading strategy, and that the actual “therapeutic” virus in the lung is leukocyte-bound in either case. Thus, autologous PBMCs isolated from a cancer patient offer a systemic route of leukocyte-mediated delivery for MYXV to cancerous metastases sites in the lung that is safe and can easily be collected from the patient.^45^^,^^46^

The data presented herein show that TNF-armed MYXV is a viable treatment strategy for metastatic osteosarcoma, as evidenced in a murine model where unarmed MYXV constructs have failed. One mechanism for this improved therapeutic benefit is the potential unique modulation of the early immune responses caused by the TNF expressed from the armed MYXV. Although usually transient in their expression, cytokines can have large, lasting effects on the immune landscape. Even within an hour after infection, many cells in the immune system will start to produce a wide profile of pro-inflammatory cytokines.^47^, ^48^, ^49^, ^50^ The cytokine expression data shortly after PBMC-mediated virotherapy of tumor-bearing mice revealed a clear change in circulating cytokine profiles between the unarmed MYXV and TNF-armed MYXV, such that we could distinguish between cytokine modulations caused by the platform MYXV alone compared to the expressed TNF transgene. We observed that uniquely TNF-responsive cytokine targets include both activators of the innate immune system, such as MCP-1, CXCL-1, IL-1α, IL-1β, and IL-6, and also adaptive immune activators including IL-2 and RANTES. These changes appear to support a more robust innate response to the tumor and promote development of anti-tumoral adaptive responses.^51^ TNF has been shown to broadly activate many classes of immune cells of both the innate and adaptive immune system. Note that the unarmed MYXV platform alone induced several unique cytokines (IL-5, eotaxin, IL-4, IFNγ, and MIP-2/CXCL-2), whereas it induced less IL-12 production when compared to either vMyx-hTNF or an untreated control. Unarmed MYXV downregulates IL-12 within 3 h, but interestingly this response switches to upregulation by the TNF-armed MYXV. This may be possibly important because IL-12 production has been shown in other studies to enhance T cell activation and effector function in combination with anti-PD-L1 therapy.^52^ Similarly, other studies have shown that, at least in non-small cell lung carcinoma, increased IL-6 production (which is also upregulated in vMyx-hTNF/PBMC-treated animals) after anti-PD-L1/anti-PD-1 therapy was a potential predictor of positive outcome in patients.^53^^,^^54^

These results taken together show that systemic delivery of TNF-armed MYXV, especially as enhanced via *ex vivo*-loaded PBMCs, is an excellent oncolytic candidate for metastatic lung osteosarcoma, particularly in combination with ICI therapy. However, broader questions do remain. The biggest among them is whether these results are applicable to all sarcomas that metastasize to the lung or whether this murine osteosarcoma model might even serve as a broader model of lung metastatic cancers as a class. Furthermore, while the TNF-armed MYXV therapy was well tolerated in mice, mice are less sensitive to the deleterious side effects of human TNF compared to humans.^55^ To bring this virus-expressed TNF therapy to the clinic, detailed toxicity studies that factor in the variable levels of expressed TNF compared to tumor burden sizes would need to be conducted in appropriately matched models. In summary, the results of this study clearly show that: (1) i.v. systemic delivery of TNF transgene-armed MYXV can be efficacious against metastatic osteosarcoma in the lung, (2) this efficacy can be further improved by *ex vivo* loading of the virus onto PBMCs prior to systemic delivery, (3) sequential multi-dosing of free virus can be comparably efficacious to single dosing of PBMC-bound virus, (4) combination therapy with ICIs can be highly effective even against established late-stage metastatic disease, and (5) the MYXV platform and the encoded TNF both contribute to the induction of a variety of cytokines important for both the innate and acquired immune responses. We conclude that, coupled with our prior work showing that *ex vivo* virotherapy with unarmed MYXV can be an effective therapeutic strategy against multiple myeloma in the BM and spleen,^31^ this systemic delivery strategy with TNF-armed MYXV can effectively treat metastatic disease in the lung. The extent to which *ex vivo* virotherapy can also be applied against other cancers in hard-to-reach tissue locations remains to be investigated.

## Materials and methods

### Cell culture, autologous carrier leukocyte collection, and viruses

K7M2-Luc cells were gifted by Dr. Lee J. Helman from the National Institutes of Health.^56^ K7M2-Luc cells were maintained in DMEM/high glucose supplemented with 10% FBS and 1% penicillin/streptomycin. Cells were maintained at 37°C and 5% carbon dioxide. Cells were subcultured in tissue culture flasks and split at a 1:6 ratio until a sufficient number of cells could be harvested. Because of initial mycoplasma contamination, cells were treated for mycoplasma using a universal mycoplasma detection kit (ATCC 30-1012K). Cells were then treated using a combination of Plasmocin (ant-mpp, InvivoGen) and Plasmocure (ant-pc, InvivoGen) for three passages verified to be mycoplasma-free. Primary BM was isolated from long bones harvested from healthy age-matched BALB/cJ donor mice. Cells were collected as previously described.^31^ PBMCs were harvested from healthy age-matched BALB/cJ mice via cardiac puncture and collected in 6.4% sodium citrate to prevent coagulation. PBMCs were isolated from whole blood using SepMate-50 from STEMCELL Technologies and Histopaque-1077 density gradient via centrifugation at 1,200 × *g* for 10 min. vMyx-135KO (used as the standard unarmed oncolytic MYXV), vMyx-11LKO (GFP-expressing knockouts of the viral M135 and M11L genes, respectively), vMyx-GFP (GFP-expressing wild-type MYXV with the GFP transgene inserted at an intergenic locus between M135 and M136 genes), vMyx-Fluc-tdTom (MYXV expressing firefly luciferase on the poxvirus synthetic early/late promoter, and tdTomato on the poxvirus late promoter P11),^57^^,^^58^ and vMyx-hTNF (GFP-expressing knockin of the human TNF gene inserted into the M131 gene) constructs were used in this study.^59^^,^^60^ The construction of vMyx-hTNF was previously described in Wang et al.^61^ Virus infection of fresh autologous BM leukocytes or PBMCs was done *ex vivo* at an MOI of 10 for 1 h at 37°C to allow for virus adsorption into the cells at a volume of 100 μL of Dulbecco’s phosphate-buffered saline (DPBS). After 1 h of cells of adsorption, virus-loaded cells were resuspended in DPBS to their final volume and infused systemically into recipient mice via retro-orbital injection.

### Animal studies

Female BALB/cJ mice were purchased from Jackson Laboratory at 5 weeks of age. Animals were acclimatized for at least 7 days prior to tumor implantation. The mice were housed in the Biodesign Institute vivarium under sterile conditions with free access to food and water during the duration of the acclimatization period and study. All housing, husbandry, and experimental protocols were done in accordance with approved IACUC protocols and institutional standards. At day 0, BALB/cJ mice were inoculated i.v. via lateral tail vein with 100 μL of DPBS containing 2 × 10^6^ K7M2-Luc tumor cells. Animals were randomized into respective cohorts at the time of first treatment. Animals that showed signs of primary tumor implantation in the tail or died prior to 2 weeks after tumor inoculation were excluded from the studies.

### Oncolytic myxoma virus treatments

Tumor-bearing animals treated with oncolytic MYXV were systemically infused with virus either after virus pre-loading *ex vivo* onto either PBMCs or BM leukocytes, or else with free (naked) virus. Animals were first anesthetized using isoflurane, and after anesthetization were given 100-μL injections of respective oncolytic virus via the retro-orbital route. Animals were then monitored for 30 min for post-injection side effects. Animals were either virus treated once (single dose) or four times (multi-dose) on the days indicated in the specific regimen.

### ICI treatments

BALB/cJ animals were inoculated with K7M2-Luc murine tumor cells as previously described in Lussier et al.^19^^,^^20^ At the treatment start date, tumor-bearing animals were treated with ICI: either anti-PD-L1 (Bio X Cell 10F.9G2), anti-PD-1 (Bio X Cell CD279), and/or anti-CTLA-4 antibody purified from UC10-4F10-11 hybridoma. Animals were treated with 10 mg/kg in a final volume of 100 μL via intraperitoneal (i.p.) injection. Animals were treated with anti-PD-L1/anti-PD-1 four or five times every third day, or with anti-CTLA-4 three times every third day.

### Cytokine analysis

BALB/cJ animals were inoculated with K7M2-Luc as previously described. At 3 days after tumor inoculation, animals were treated with saline, unarmed vMyx-GFP/PBMCs, or TNF-armed vMyx-hTNF/PBMCs. Virus infusion time is defined as time 0. At 1, 3, and 24 h after virus treatment, whole blood was collected via cardiac puncture. Blood was collected in BD Microtainer tubes and centrifuged at 3,000 rpm. Serum was stored at −80°C. For multiplex cytokine analysis, serum was assayed for the following murine cytokine levels by ELISA (at Boster Biological Technology): IL-1α, IL-1β, IL-2, IL-3, IL-4, IL-5, IL-6, IL-10, IL-12p70, IL-17, MCP-1(CCL-2), IFNγ, TNF-α, MIP-1α (CCL3), GM-CSF, RANTES (CCL5), eotaxin, MIP-2 (CXCL2), KC (CXCL1), MDC (CCL22), TARC (CCL17), TCA3 (CCL1), and IL-13. The screen also included the human versions of TNF-α and TNF-β (lymphotoxin-α). The full results of the cytokine screening are indicated in Figure S1.

### *In vivo* imaging of K7M2-Luc tumors

Tumor progression was assessed using a PerkinElmer IVIS Lumina III system. Animals were i.p. injected with 100 μL of d-luciferin suspended in DPBS (30 mg/mL). Animals were then sedated using isoflurane and were imaged for 1 min using the IVIS Lumina III system. Following imaging, tumor luminescence levels were measured using Caliper Life Sciences Live Image v4.5. Tumor signals were measured for 95% of radiance using the program’s automatic drawing application and were usually first detectable in >95% of control mice by approximately 1–2 weeks after tumor implantation. In untreated tumor-inoculated mice, the acquisition of 5 × 10^5^ radiance units from the lung was defined as established late-stage disease, which was used as the criteria for inclusion in the cohorts described in Figure 4. Generally, endpoint euthanasia criteria were met after 10^8^ radiance units were detected in the lung. Euthanasia criteria were assessed based a combination of on the animals breathing, energy level (lethargy), and ability to ambulate/neurological symptoms.

### Statistical analysis

Tumor growth was determine using radiance units defined as photons/s/cm^2^/steradian using an automatically determined region of interest (ROI) based on a threshold of a minimum of 5% of peak photon intensity. Statistical analysis for this study was done using GraphPad Prism. Differences for Kaplan-Meier survival curves were determined using log-rank tests. Differences in tumor radiance determined by imaging and in cytokine expression was determined by unpaired t tests.

## Supplemental information

Document S1. Figures S1 and S2

Document S2. Article plus supplemental information
