# Supplementary material for: Systemic delivery of TNF-armed myxoma virus plus immune checkpoint inhibitor eliminates lung metastatic mouse osteosarcoma
Source: Mol Ther Oncolytics. 2021 Aug 6;22:539–54. doi: 10.1016/j.omto.2021.07.014 (PMC8433070; doi:10.1016/j.omto.2021.07.014)
Supplement: Document S1. Figures S1 and S2 [file mmc1.pdf]

OMTO, Volume *22*

Supplemental information

Systemic delivery of TNF-armed myxoma virus plus immune checkpoint inhibitor eliminates lung metastatic mouse osteosarcoma

John D. Christie, Nicole Appel, Hannah Canter, Jazmin Galvan Achi, Natalie M. Elliott, Ana Lemos de Matos, Lina Franco, Jacquelyn Kilbourne, Kenneth Lowe, Masmudur M. Rahman, Nancy Y. Villa, Joshua Carmen, Evelyn Luna, Joseph Blattman, and Grant McFadden

Supplemental Figure 1

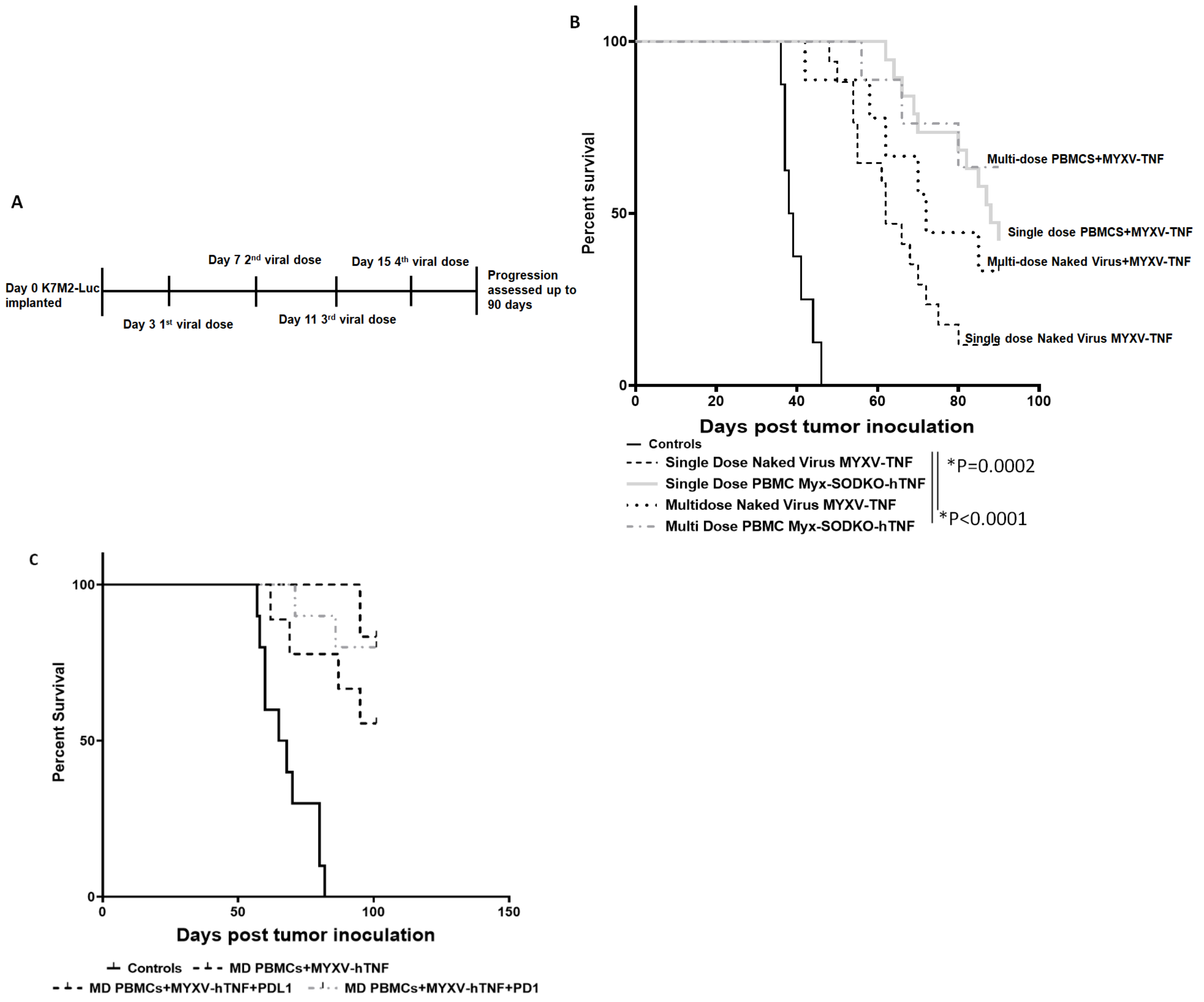

Supplemental Figure 1

Multi-dosing of systemic TNF-armed MYXV/PBMCs vs free virus compared to single dose regimen.

(A) Balb/c mice were inoculated with K7M2-Luc cells at day 0. At day 3, animals received armed MYXV-TNF systemically as free virus or as *ex vivo* loaded MYXV-TNF/PBMCs. Animals in the multi-dosing groups then received 3 subsequent doses every fourth day following the first dose, for a total of 4 doses. (B)Meier-Kaplan survival curves comparing single dose vs multiple doses of either naked virus systemic vMyx-TNF or vMyx-TNF/PBMCs. All treatments lead to a significant increase in survival than animals that were left untreated. Both multi-dose regimens showed trends towards increased mean survival compared to respective single dose regimens, however, was not statistically different. (C) Experimental setup is show in Fig 3A. Meier-Kaplan survival curves comparing multidose vMyx-TNF/PBMCs vs multidose vMyx-TNF/PBMCs plus either anti-PD-1 or anti-PD-L1 therapy. Both combination therapies showed trends towards increased mean survival, however, were not significantly different than vs multidose vMyx-TNF/PBMCs alone.

Supplemental Figure 2

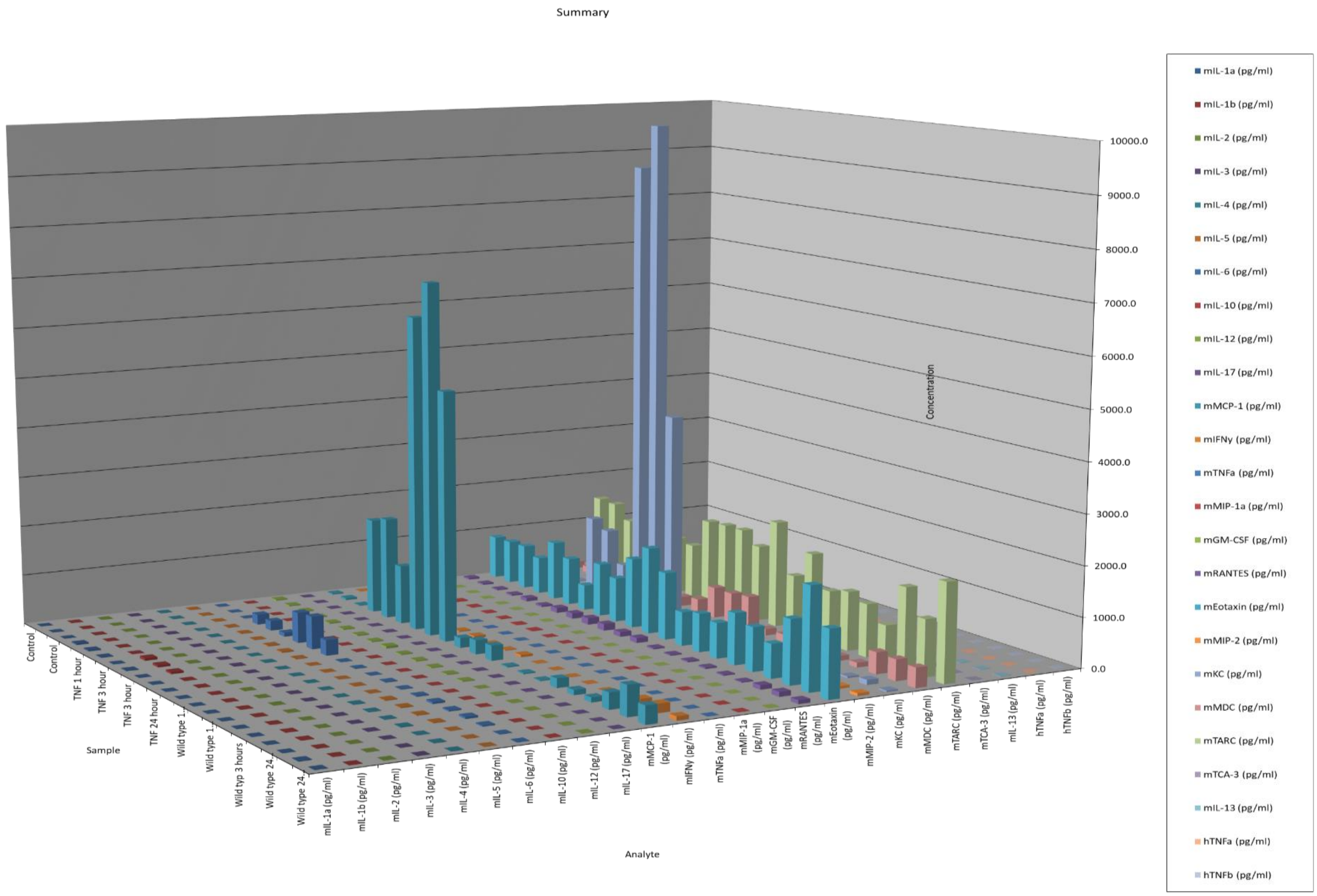

Supplemental Figure 2

Measurement and comparison of total cytokine production across all treatments, animals and time points.

Balb/c mice were inoculated with K7M2-Luc cells at day 0. At day 3, animals were either left untreated, systemically infused with unarmed vMyx-GFP/PBMC (MYXV), or armed vMyx-hTNF/PBMC (MYXV-TNF). Serum was collected at 1 hour, 3 hours or 24 hours post virus treatment. The graph shows all cytokines assayed and their overall circulating amounts in ng/pg per ml in individual animals at 1, 3 and 24 hours post treatment.
